# Evaluation of Sudden Cardiac Arrest by Race/Ethnicity Among Residents of Ventura County, California, 2015-2020

**DOI:** 10.1001/jamanetworkopen.2021.18537

**Published:** 2021-07-29

**Authors:** Kyndaron Reinier, Arayik Sargsyan, Harpriya S. Chugh, Kotoka Nakamura, Audrey Uy-Evanado, Damon Klebe, Robert Kaplan, Katy Hadduck, Daniel Shepherd, Christopher Young, Angelo Salvucci, Sumeet S. Chugh

**Affiliations:** 1 Center for Cardiac Arrest Prevention, Smidt Heart Institute, Cedars-Sinai Health System, Los Angeles, California; 2 Department of Epidemiology and Population Health, Albert Einstein College of Medicine, Bronx, New York; 3 Ventura County Health Care Agency, Ventura, California; 4 Ventura County Medical Examiner’s Office, Ventura, California

## Abstract

**Importance:**

Sudden cardiac arrest (SCA) is a major public health problem. Owing to a lack of population-based studies in multiracial/multiethnic communities, little information is available regarding race/ethnicity-specific epidemiologic factors of SCA.

**Objective:**

To evaluate the association of race/ethnicity with burden, outcomes, and clinical profile of individuals experiencing SCA.

**Design, Setting, and Participants:**

A 5-year prospective, population-based cohort study of out-of-hospital SCA was conducted from February 1, 2015, to January 31, 2020, among residents of Ventura County, California (2018 population, 848 112: non-Hispanic White [White], 45.8%; Hispanic/Latino [Hispanic], 42.4%; Asian, 7.3%; and Black, 1.7% individuals). All individuals with out-of-hospital SCA of likely cardiac cause and resuscitation attempted by emergency medical services were included.

**Exposures:**

Data on circumstances and outcomes of SCA from prehospital emergency medical services records and data on demographics and pre-SCA clinical history from detailed archived medical records, death certificates, and autopsies.

**Main Outcomes and Measures:**

Annual age-adjusted SCA incidence by race and ethnicity and SCA circumstances and outcomes by ethnicity. Clinical profile (cardiovascular risk factors, comorbidity burden, and cardiac history) by ethnicity, overall, and stratified by sex.

**Results:**

A total of 1624 patients with SCA were identified (1059 [65.2%] men; mean [SD] age, 70.9 [16.1] years). Race/ethnicity data were available for 1542 (95.0%) individuals, of whom 1022 (66.3%) were White, 381 (24.7%) were Hispanic, 86 (5.6%) were Asian, 31 (2.0%) were Black, and 22 (1.4%) were other race/ethnicity. Annual age-adjusted SCA rates per 100 000 residents of Ventura County were similar in White (37.5; 95% CI, 35.2-39.9), Hispanic (37.6; 95% CI, 33.7-41.5; *P* = .97 vs White), and Black (48.0; 95% CI, 30.8-65.2; *P* = .18 vs White) individuals, and lower in the Asian population (25.5; 95% CI, 20.1-30.9; *P* = .006 vs White). Survival to hospital discharge following SCA was similar in the Asian (11.8%), Hispanic (13.9%), and non-Hispanic White (13.0%) (*P* = .69) populations. Compared with White individuals, Hispanic and Asian individuals were more likely to have hypertension (White, 614 [76.3%]; Hispanic, 239 [79.1%]; Asian, 57 [89.1%]), diabetes (White, 287 [35.7%]; Hispanic, 178 [58.9%]; Asian, 37 [57.8%]), and chronic kidney disease (White, 231 [29.0%]; Hispanic, 123 [40.7%]; Asian, 33 [51.6%]) before SCA. Hispanic individuals were also more likely than White individuals to have hyperlipidemia (White, 380 [47.2%]; Hispanic, 165 [54.6%]) and history of stroke (White, 107 [13.3%]; Hispanic, 55 [18.2%]), but less likely to have a history of atrial fibrillation (White, 251 [31.2%]; Hispanic, 59 [19.5%]).

**Conclusions and Relevance:**

The results of this study suggest that the burden of SCA was similar in Hispanic and White individuals and lower in Asian individuals. The Asian and Hispanic populations had shared SCA risk factors, which were different from those of the White population. These findings underscore the need for an improved understanding of race/ethnicity-specific differences in SCA risk.

## Key Points

**Question:**

Are race and ethnicity associated with incidence rates, clinical profiles, and outcomes in individuals experiencing sudden cardiac arrest?

**Findings:**

In this 5-year cohort study of sudden cardiac arrest in a Southern California community comprising 848 112 individuals, rates of sudden cardiac arrest were similar in the non-Hispanic White, non-Hispanic Black, and Hispanic populations, but lower in the Asian population. Survival outcomes were similar by race and ethnicity, despite a higher prevalence before sudden cardiac arrest of hypertension, diabetes, and chronic kidney disease in Asian and Hispanic individuals compared with White individuals.

**Meaning:**

The findings of this study suggest that sudden cardiac arrest prevention in the overall population may require an improved understanding of race/ethnicity-specific differences in risk.

## Teaser

This cohort study examines the incidence of sudden cardiac arrest and risk factors by race/ethnicity in residents of Ventura County, California.

## Introduction

Out-of-hospital sudden cardiac arrest (SCA), a sudden loss of the pulse, affects approximately 350 000 individuals in the US annually.^1^ Despite resuscitation efforts, the mortality rate exceeds 90%, thereby making prediction and prevention of this condition a major priority.^1^ The burden of SCA is affected by multiple factors, including race, socioeconomic status (SES), and potentially race/ethnicity. For example, population-based studies of the US Black population consistently report a 2-fold higher incidence rate compared with their White counterparts,^2,3,4,5^ and there is an association between low SES and increased SCA annual incidence.^6,7^ The Hispanic/Latino population is the largest and most rapidly growing racial/ethnic minority group in the US, but, to our knowledge, there have been no prospective studies evaluating SCA burden for this prominent population.

Studies of sudden cardiac death (SCD) in the US performed more than 2 decades ago suggested that the burden of this condition was significantly lower in Hispanic/Latino and Asian individuals compared with non-Hispanic White and Black individuals.^4,8^ However, these retrospective studies identified SCD from death certificates, which is a relatively inaccurate method yielding a positive predictive value in the range of 19%.^9^ Also, identification of SCA survivors, constituting approximately 10% of all cases, needs prospective ascertainment. Contemporary data from Korea^10^ and Japan^11^ suggest that rates among Asian individuals in those countries are lower than in the US overall, but data on SCA incidence among the Asian American population are lacking. Therefore, a contemporary multiethnic assessment of SCA is warranted. We conducted a prospective cohort study of SCA among all residents of Ventura County, California, a US community in which more than 40% of the residents are Hispanic.

## Methods

Individuals with SCA were identified from the Ventura Prediction of Sudden Death in Multi-ethnic Communities (PRESTO) Study, an ongoing investigation of all out-of-hospital SCAs in Ventura County, California (2018 population, 848 112) from February 1, 2015, to January 31, 2020. All incident cases of presumed SCA were prospectively identified through collaboration with the county’s 2-tiered emergency medical services (EMS) system. Patients with resuscitation attempted by EMS were subjected to detailed adjudication, concurrent with the evolution of data. Only residents of Ventura County were included. All existing records were obtained and reviewed for each patient, including the EMS prehospital care report, medical records from the region’s hospitals (including records from the index SCA event and pre-SCA clinical history), death certificates from California state vital statistics records, and the medical examiner report, including autopsy if available. Based on this detailed information, 3 physicians (A.U.-E., A. Sargsyan, and S.S.C.) performed in-house adjudication for determination of SCA, which was defined as a sudden, unexpected, pulseless condition of likely cardiac origin.^1^ All patients with an identifiable noncardiac cause for cardiac arrest were excluded, such as trauma, drug overdose, and chronic terminal illness (eg, cancer not in remission). Primary adjudicators (A.U.-E. and A. Sargsyan) reviewed records independently from the study designers (K.R. and S.S.C.). This study design was based on methods used in the Oregon Sudden Unexpected Death Study, a population-based study ongoing for 18 years.^9,12^ The PRESTO Study was approved by the institutional review boards of Ventura County Medical Center, Cedars-Sinai Health System, and all participating hospitals and health systems. All survivors of cardiac arrest provided written informed consent; for nonsurvivors, this requirement was waived. This study followed the Strengthening the Reporting of Observational Studies in Epidemiology (STROBE) reporting guideline for cohort studies.^13^

Race and ethnicity data for individual patients were obtained from death certificates, medical examiner reports, medical records, and EMS prehospital care reports. US Census data were used for the race/ethnic composition of the community. Race and ethnicity were categorized as non-Hispanic White (White), Black/African American (Black), Hispanic/Latino (Hispanic), Asian, and other. Hispanic included any individual reporting Hispanic ethnicity, regardless of race. Other included American Indian/Alaska Native and Hawaiian/Pacific Islander. Demographic characteristics and circumstances related to the cardiac arrest event were obtained from the EMS prehospital care report and characterized using the Utstein template.^14^ To determine the outcome of SCA for each patient, we reviewed the EMS record and/or medical examiner report/death certificate, if applicable, and all available hospital records. Socioeconomic status was evaluated by 2 indicators: median household income and educational level. Each patient was assigned the median household income of their residential census tract, based on US Census 2018 estimates for Ventura County, and analyzed as quartiles. Individual educational achievement was obtained from the death certificate for individuals who did not survive and from an in-person interview for survivors. The clinical profile of patients was assembled from medical records, as described in detail previously.^9,12,15^ Comorbidities and history of cardiovascular events were determined from physician-noted health history in medical records. Results from autopsies were obtained from the Ventura County medical examiner.

### Statistical Analysis

For calculation of crude SCA rates, incidence counts based on age and race/ethnicity were used as the numerator, and US Census 2018 5-year estimates of the Ventura County population by age and race/ethnicity were used as the denominator. Age-adjusted incidence was calculated using US Census 2015 estimates of the standard population within each age and race/ethnicity category, using SAS, version 9.4 (SAS Institute Corp) general linear models. Comparisons of resuscitation and pre-SCA clinical profile were limited to Asian, Hispanic, and White patients with SCA owing to the small numbers in the other groups. The comparisons were performed using logistic regression models with race/ethnicity as the categorical independent variable, adjusted for age as a continuous variable to evaluate the age-adjusted association of race/ethnicity with each arrest circumstance or clinical finding. For continuous variables (age and response time in minutes), an analogous approach with analysis of variance was used, with Tukey post hoc tests for pairwise comparisons by race/ethnicity. In addition, we evaluated sex-specific differences in clinical profile among Hispanic and White individuals using χ^2^ tests. A 2-sided, unpaired value of *P* < .05 was considered significant.

## Results

**Figure 1.**
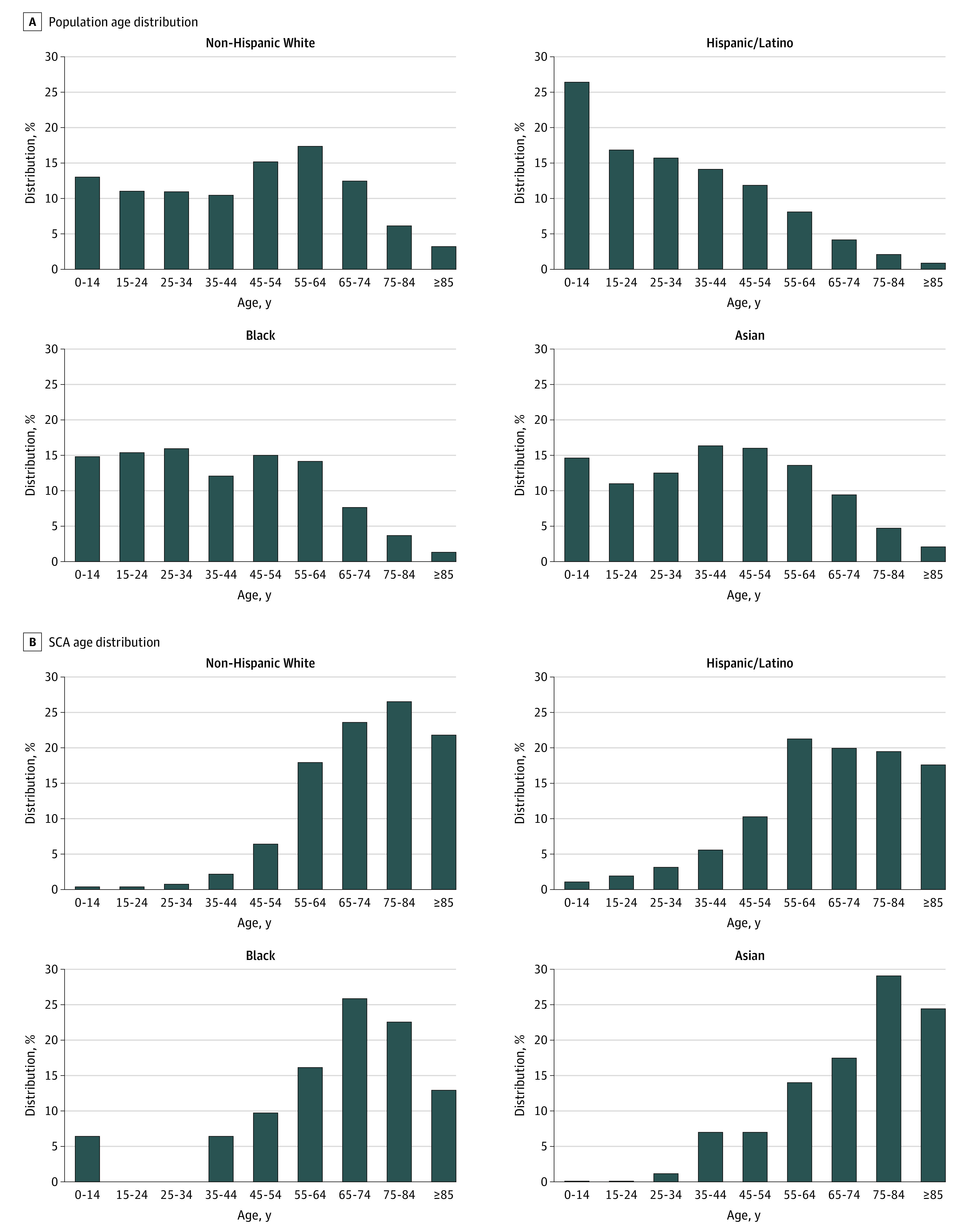
Ventura County Population and Patients With Sudden Cardiac Arrest (SCA) by Race and Ethnicity US Census 2018 data were used for the race/ethnic composition of the community.

From February 1, 2015, to January 31, 2020, a total of 1624 SCAs were identified and adjudicated, of whom 1059 (65.2%) were men and 565 (34.8%) were women, and the overall mean (SD) age was 70.9 (16.1) years. A total of 45 (2.8%) individuals were younger than 35 years, 486 (30.0%) were aged 35 to 64 years, 763 (47.0%) were aged 65 to 84 years, and 329 (20.3%) were 85 years or older. Data on race/ethnicity were available for 1542 (95.0%) individuals with SCA. Of these, 1022 (66.3%) were White, 381 (24.7%) Hispanic, 86 (5.6%) Asian, and 31 (2.0%) Black individuals, and 22 (1.4%) were other race/ethnicity. As a comparison, the race/ethnicity distributions of the 2018 population of Ventura County were 45.8% White, 42.4% Hispanic, 7.3% Asian, and 1.7% Black individuals. The mean (SD) age at the time of SCA in Hispanic (67.0 [18.3] years) and Black (65.6 [22.2] years) individuals was approximately 6 years younger than in White (72.6 [14.5] years) and Asian (73.6 [16.1] years) individuals (*P* < .001). Figure 1 illustrates differences in the age distribution by race and ethnicity of patients with SCA as well as the overall population of Ventura County; the age distribution of patients with SCA at least partly mirrored the younger age distribution in the Hispanic and Black populations compared with the White and Asian populations.

**Figure 2.**
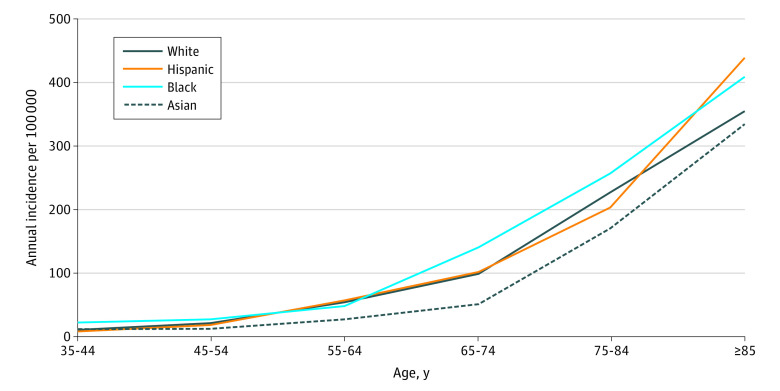
Annual Sudden Cardiac Arrest Incidence Rates by Race and Ethnicity in Ventura County, California Lines show annual age-specific incidence of sudden cardiac arrest per 100 000 in each race/ethnic group.

The overall annual incidence of SCA in Ventura County was 36.3 per 100 000 residents, and when adjusted for age, was 36.5 per 100 000 residents (95% CI, 34.6-38.3 per 100 000 residents). Crude rates were 52.6 per 100 000 residents among White, 21.2 per 100 000 residents among Hispanic, 41.8 per 100 000 residents among Black, and 27.8 per 100 000 residents among Asian individuals, partly reflecting the different population age distributions in each race and ethnic group (Figure 1). In Ventura County, 93% of the Hispanic population was age 65 years or younger, and 43% of Hispanic individuals with SCA were younger than 65 years. Seventy-eight percent of the county’s White population was aged 65 years or younger, and 26% of White individuals with SCA were younger than 65 years. Annual age-adjusted rates per 100 000 residents were similar in White (37.5; 95% CI, 35.2-39.9) and Hispanic (37.6; 95% CI, 33.7-41.5; *P* = .97) individuals; the rate was somewhat higher among Black individuals, but the difference was not significant (48.0; 95% CI, 30.8-65.2; *P* = .18 vs White), and the rate was lower among Asian individuals (25.5; 95% CI, 20.1-30.9; *P* = .006 vs White). Patients with SCA who were younger than 35 years comprised a small percentage of the total SCAs (1.6% among White, 6.0% among Hispanic, 6.5% among Black, and 1.2% among Asian individuals). Age-specific rates in each race and ethnic group (Figure 2) show that at most ages, White and Hispanic individuals had similar rates, Black individuals had somewhat higher rates, and Asian individuals had somewhat lower rates.

Sudden cardiac arrest circumstances and outcomes overall were similar across race/ethnicity (Table 1). Proportions with witnessed SCA and receipt of bystander cardiopulmonary resuscitation (CPR), as well as EMS response time, were comparable between the Asian, Hispanic, and White individuals with SCA. Asian individuals were 2.5 to 3 times more likely than White individuals to present with a nonshockable (pulseless electrical activity/asystole) than a shockable (ventricular fibrillation/tachycardia) rhythm; Hispanic individuals were somewhat more likely than White individuals to present with asystole. All 3 groups were similarly likely to have return of spontaneous circulation following resuscitation and to survive to hospital discharge (11.8% Asian, 13.9% Hispanic, and 13.0% White individuals; *P* = .69) (Table 1). Similar survival outcomes by race/ethnicity were also observed when stratified by presenting rhythm (Table 1).

**Table 1.** Resuscitation Circumstances and Outcomes by Race/Ethnicity in Patients With SCA^a^

Variable	No. (%)			Odds ratio (95% CI)^b^	
	Asian (n = 86)	Hispanic (n = 381)	Non-Hispanic White (n = 1022)	Asian vs non-Hispanic White	Hispanic vs non-Hispanic White
Age, mean (SD), y	73.6 (16.1)	67.0 (18.3)	72.6 (14.5)	0.9 (−3.2 to 5.1)^c^	−5.6 (−7.8 to −3.4)^c^
Sex					
Female	40 (46.5)	129 (33.9)	350 (34.2)	1.7 (1.1 to 2.6)	1.1 (0.8 to 1.4)
Male	46 (53.5)	252 (66.1)	672 (65.8)	0.6 (0.4 to 1.0)	0.9 (0.7 to 1.2)
Witnessed status					
Bystander witnessed	36 (41.9)	147 (38.9)	391 (38.6)	1.1 (0.7 to 1.8)	0.98 (0.8 to 1.3)
EMS witnessed	7 (8.1)	42 (11.1)	112 (11.0)	0.7 (0.3 to 1.7)	1.0 (0.7 to 1.5)
Not witnessed	43 (50.0)	189 (50.0)	511 (50.4)	1 [Reference]	1 [Reference]
Missing	0	3	8		
SCA location					
Home	57 (69.5)	286 (80.6)	732 (76.4)	1 [Reference]	1 [Reference]
Public	4 (4.9)	12 (3.4)	33 (3.4)	1.6 (0.5 to 4.6)	0.8 (0.4 to 1.5)
ED/outpatient clinic	6 (7.3)	19 (5.4)	21 (2.2)	3.7 (1.4 to 9.6)	2.1 (1.1 to 4.0)
Care facility	14 (17.1)	33 (9.3)	161 (16.8)	1.0 (0.6 to 1.9)	0.6 (0.4 to 0.9)
Other	1 (1.2)	5 (1.4)	11 (1.1)	NA^d^	NA^d^
Missing	4	26	64	NA	NA
Bystander CPR	54 (62.8)	196 (51.4)	563 (55.1)	1.4 (0.9 to 2.2)	0.8 (0.7 to 1.1)
Response time					
Mean (SD), min	6.4 (3.7)	6.0 (4.5)	6.2 (3.8)	0.2 (−0.8 to 1.3)^e^	−0.2 (−0.7 to 0.4)^e^
No.	86	380	1012		
>4 min	73 (84.9)	299 (78.7)	834 (82.4)	1.2 (0.7 to 2.2)	0.8 (0.6 to 1.1)
Missing	0	1	10	NA	NA
Presenting rhythm					
VF/VT	10 (11.6)	91 (23.9)	267 (26.2)	1 [Reference]	1 [Reference]
PEA	34 (39.5)	112 (29.5)	305 (29.9)	3.1 (1.5 to 6.5)	1.36 (1.0 to 1.9)
Asystole	40 (46.5)	175 (46.1)	441 (43.3)	2.5 (1.2 to 5.2)	1.4 (1.1 to 2.0)
Other	2 (2.3)	2 (0.5)	6 (0.6)	NA	NA
Missing	0	1	3	NA	NA
ROSC^f^					
Overall	25 (29.4)	119 (31.5)	269 (26.6)	1.2 (0.7 to 1.9)	1.1 (0.9 to 1.5)
With VF/VT	5 (50.0)	51 (56.0)	134 (50.6)	0.9 (0.3 to 3.3)	1.0 (0.6 to 1.7)
With PEA or asystole	18 (24.7)	67 (23.5)	130 (17.6)	1.5 (0.9 to 2.7)	1.4 (1.0 to 1.9)
Missing	1	3	12	NA	NA
STHD ^g^					
Overall	10 (11.8)	53 (13.9)	133 (13.0)	0.9 (0.4 to 1.8)	0.8 (0.5 to 1.1)
With VF/VT	5 (50.0)	36 (39.6)	93 (35.0)	1.7 (0.5 to 6.2)	0.9 (0.5 to 1.5)
With PEA or asystole	4 (5.4)	16 (5.6)	36 (4.9)	1.1 (0.4 to 3.3)	1.0 (0.5 to 1.8)
Missing	1	1	9	NA	NA

Abbreviations: CPR, cardiopulmonary resuscitation; ED, emergency department; EMS, emergency medical services; NA, not applicable; PEA, pulseless electrical activity; ROSC, return of spontaneous circulation; SCA, sudden cardiac arrest; STHD, survival to hospital discharge; VF/VT, ventricular fibrillation/ventricular tachycardia.

^a^ For variables with missing values, proportions were calculated using the nonmissing data as the denominator. For logistic regression and analysis of variance, we performed a complete case analysis excluding individuals with missing data.

^b^ Except where noted otherwise, data were determined with separate multivariate logistic regression models with each variable in the table as the outcome and race/ethnicity as an independent variable, adjusted for age.

^c^ Calculated from analysis of variance with Tukey post hoc test for pairwise comparisons.

^d^ Excluded from logistic regression model owing to small numbers.

^e^ Calculated from analysis of covariance adjusted for age, with Tukey post hoc test for pairwise comparisons.

^f^ Overall was calculated for all SCA cases, regardless of presenting rhythm; in the VF/VT and PEA or asystole rows, ROSC was calculated by presenting rhythm, but not calculated for those with other presenting rhythms.

^g^ Overall was calculated for all SCA cases, regardless of presenting rhythm; in the VF/VT and PEA or asystole rows, STHD was calculated by presenting rhythm, but not calculated for those with other presenting rhythms.

Hispanic individuals were more likely than White individuals to live in a census tract with below-median household income (72% vs 46%; *P* < .001) and less likely to have completed education beyond high school (11% vs 39%; *P* < .001). Income and educational levels in the Asian and White populations were similar (*P* ≥ .12).

A detailed clinical profile was available from archived medical records for 302 (79.3%) of the Hispanic, 805 (78.8%) of the White, and 64 (74.4%) of the Asian individuals, and individuals aged 18 years or older were included in the comparisons of clinical history (Table 2). Diabetes was more prevalent among the Asian (37 [57.8%]) and Hispanic (178 [58.9%]) vs the White (287 [35.7%]) individuals with SCA. Chronic kidney disease was substantially more common among Asian (33 [51.6%]) and Hispanic (123 [40.7%]) than White (231 [29.0%]) individuals. Hypertension was also more prevalent in Asian (57 [89.1%]) and Hispanic (239 [79.1%]) than White (614 [76.3%]) individuals. A history of smoking was less common among Asian (4 of 58 [6.9%]) and Hispanic (34 of 256 [13.3%]) individuals than among White (103 of 647 [15.9%]) individuals. Although a history of atrial fibrillation was less common among Hispanic (59 [19.5%]) than among White (251 [31.2%]) individuals, a history of stroke was substantially more common (Hispanic, 55 [18.2%] vs White, 107 [13.3%]). Despite the difference in cardiovascular risk factor burden, the prevalence of recognized coronary artery disease before SCA was similar across the 3 groups, ranging from 43% to 48%.

**Table 2.** Clinical Profile by Race/Ethnicity in Patients With SCA^a^

Variable	No. (%)			Odds ratio (95% CI)^b^	
	Asian (n = 64)	Hispanic (n = 302)	Non-Hispanic White (n = 805)	Asian vs non-Hispanic White	Hispanic vs non-Hispanic White
Age, mean (SD)	73.2 (17.2)	69.1 (16.6)	73.6 (13.5)	−0.4 (−4.9 to 4.0)^c^	−4.4 (−6.7 to −2.1)^c^
Male	36 (56.3)	196 (64.9)	523 (65.0)	0.7 (0.4 to 1.2)	0.9 (0.7 to 1.2)
Hypertension	57 (89.1)	239 (79.1)	614 (76.3)	2.8 (1.2 to 6.3)	1.4 (1 to 2.0)
Hyperlipidemia	37 (57.8)	165 (54.6)	380 (47.2)	1.6 (0.9 to 2.6)	1.5 (1.1 to 1.9)
Obesity	17 (28.8)	118 (43.2)	259 (38.8)	0.6 (0.3 to 1.1)	1.1 (0.8 to 1.4)
Unknown	5	29	137		
Smoking	4 (6.9)	34 (13.3)	103 (15.9)	0.3 (0.1 to 0.9)	0.6 (0.4 to 1.0)
Unknown	6	46	158		
Diabetes	37 (57.8)	178 (58.9)	287 (35.7)	2.5 (1.5 to 4.2)	2.6 (2.0 to 3.4)
COPD	11 (17.2)	43 (14.2)	191 (23.7)	0.7 (0.3 to 1.3)	0.6 (0.4 to 0.8)
Asthma	3 (4.7)	18 (6.0)	62 (7.7)	0.6 (0.2 to 1.9)	0.7 (0.4 to 1.2)
Chronic kidney disease	33 (51.6)	123 (40.7)	231 (29.0)	2.7 (1.6 to 4.5)	1.8 (1.4 to 2.4)
History of MI	12 (18.8)	48 (15.9)	140 (17.4)	1.1 (0.6 to 2.1)	1.0 (0.7 to 1.4)
Documented CAD	31 (48.4)	137 (45.4)	346 (43.0)	1.3 (0.8 to 2.1)	1.2 (0.9 to 1.6)
Heart failure	24 (37.5)	97 (32.1)	238 (29.6)	1.4 (0.8 to 2.5)	1.3 (0.9 to 1.7)
History of atrial fibrillation	19 (29.7)	59 (19.5)	251 (31.2)	0.9 (0.5 to 1.6)	0.6 (0.4 to 0.8)
History of stroke	10 (15.6)	55 (18.2)	107 (13.3)	1.2 (0.6 to 2.4)	1.6 (1.1 to 2.3)

Abbreviations: CAD, coronary artery disease; COPD, chronic obstructive pulmonary disease; MI, myocardial infarction; SCA, sudden cardiac arrest.

^a^ Analysis limited to individuals aged 18 years or older with pre-SCA medical records available. For variables with missing values, proportions were calculated using the nonmissing data as the denominator. For logistic regression and analysis of variance, a complete case analysis excluding individuals with missing data was performed.

^b^ Except where noted otherwise, data were determined with separate multivariate logistic regression models with each variable in the table as the outcome and race/ethnicity as a predictor, adjusted for age.

^c^ Calculated from analysis of variance with Tukey post hoc test for pairwise comparisons.

**Figure 3.**
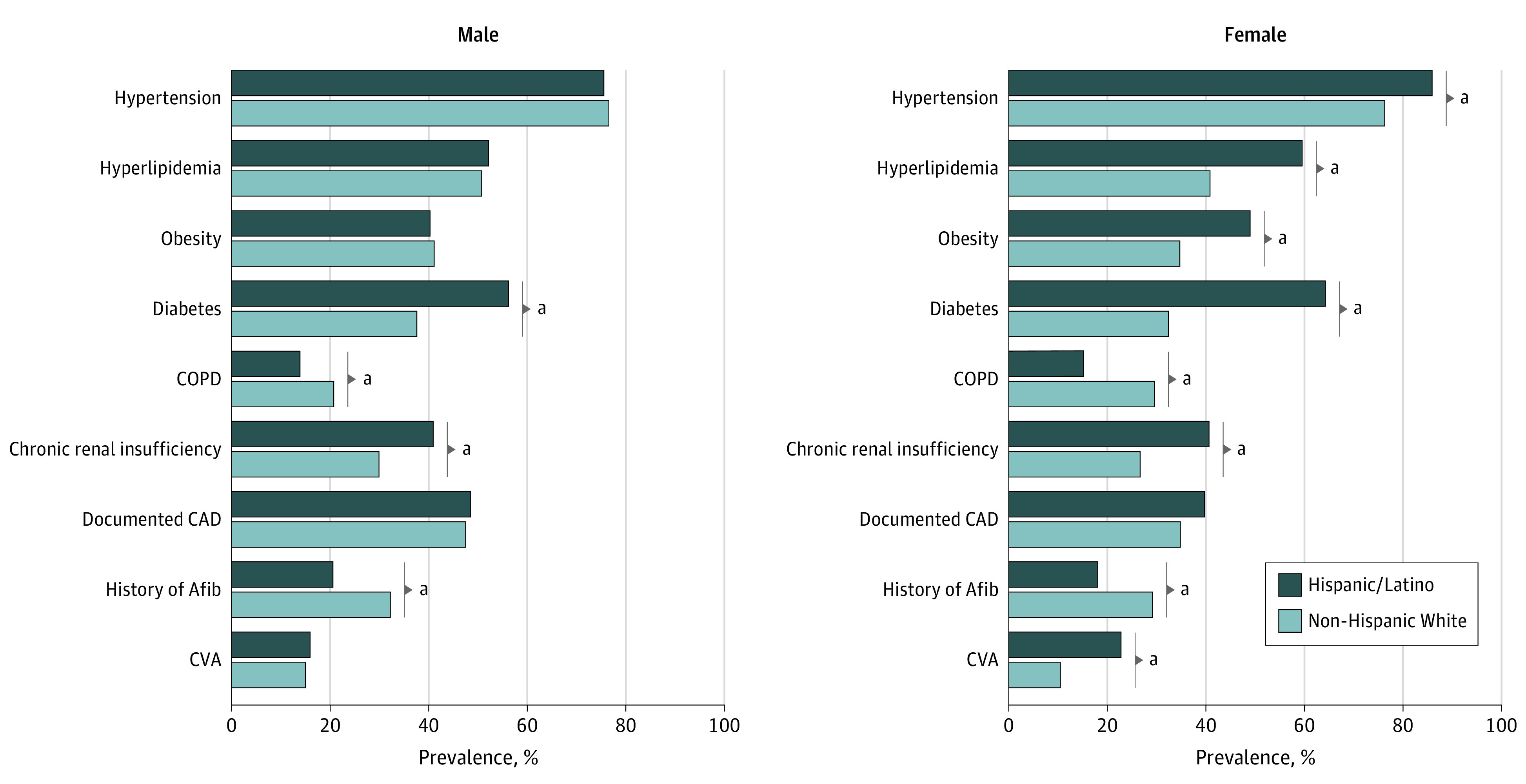
Sex-Specific Clinical Profile in Hispanic and Non-Hispanic White Patients With Sudden Cardiac Arrest (SCA) in Ventura County, California Afib indicates atrial fibrillation; CAD, coronary artery disease; COPD, chronic obstructive pulmonary disease; CVA, cerebrovascular accident. ^a^Significant differences (*P* < .05) by ethnicity.

When stratified by sex, the overall ethnic differences in diabetes and chronic kidney disease (higher in Hispanics) and COPD and atrial fibrillation (lower in Hispanics) were observed in both men and women (Figure 3). However, other ethnic differences in risk factors were sex specific. Among women only, Hispanic compared with White women were more likely to have hypertension (91 [86%] vs 215 [76%];* P* = .03), hyperlipidemia (63 [59%] vs 115 [41%];* P* = .001), and obesity (46 [49%] vs 81 [35%]; *P* = .002) (Figure 3), and to have a history of stroke (24 [23%] vs 29 [10%]; *P* = .002) at the time of their SCA.

## Discussion

The lack of contemporary, prospective, population-based studies of SCA in multiethnic communities represents a substantial knowledge gap in the field. These findings from the ongoing PRESTO study in Ventura County, California, indicate that annual age-adjusted SCA rates were similar in White and Hispanic individuals, and lower in Asian individuals. There were no significant differences identified between Asian, White, and Hispanic patients for emergency services response or SCA survival outcomes. In contrast to the similar rates and outcomes observed, Hispanic residents had significantly lower SES and a clinical profile typically associated with higher cardiovascular risk. Asian and Hispanic patients with SCA were significantly more likely than White patients with SCA to have a history of hypertension, diabetes, and chronic kidney disease. Hispanic individuals with SCA were less likely to have a history of atrial fibrillation but more likely to have a history of stroke. Hispanic women were also more likely to have a history of hyperlipidemia and obesity compared with White women with SCA.

Although we found similar age-adjusted rates in the Hispanic and White populations, earlier studies reported lower rates among Hispanic individuals.^4,8^ For example, age-adjusted rates of SCD occurring out-of-hospital or in emergency departments in 40 US states from 1980 to 1985 were much lower in Hispanic individuals (men, 75 per 100 000; women, 35 per 100 000) compared with White individuals (men, 166 per 100 000; women, 74 per 100 000).^8^ Similarly, based on US vital statistics mortality data from 1989 to 1998, the age-adjusted rate of SCD among the Hispanic population was just over half the rate among non-Hispanic groups.^4^ However, SCA ascertainment in these previously published studies was not prospective, relied on death certificate data, and was conducted at least 2 decades ago—all factors that may explain the higher SCD rates compared with the present study.^9^ A more recent study conducted in San Francisco in 2012 included all patients with SCD evaluated by the medical examiner; only those with arrhythmic causes were included in rate calculations.^16^ Although the study had a small number of Hispanic individuals (21 cases of SCA, 11 with arrhythmic causes), their results were similar to ours: the annual rate of arrhythmic SCD in Hispanic individuals (13.5 per 100 000) was not significantly different (*P* = .40) than the rate in White individuals (20.1 per 100 000).

Our study found lower age-adjusted SCA rates among Asian compared with White individuals. These findings are consistent with reports of substantially lower SCA incidence from studies in Korea^10^ and Japan.^11^ However, results from the San Francisco study indicated that Asian residents of San Francisco (18.9 per 100 000) had an annual SCA rate similar to that of White residents (*P* = .90).^16^

In the present study, 3 established, independent factors associated with risk of SCA (ie, diabetes, hypertension, and chronic kidney disease) were significantly more common among Asian and Hispanic individuals with SCA compared with White individuals. Hispanic individuals were also more likely to have hyperlipidemia and stroke. Among Hispanic women, the differences in hypertension, hyperlipidemia, and history of stroke were more pronounced than among men, and Hispanic women were also more likely to be obese compared with their White counterparts. Despite the higher cardiovascular risk profile in the Asian and Hispanic cohorts, all groups had a similar prevalence of recognized coronary artery disease (CAD) diagnosed prior to SCA. These findings suggest race/ethnicity-specific areas of focus for education and preventive interventions that may improve screening and early diagnosis to reduce SCA burden. The finding of a lower prevalence of atrial fibrillation among Hispanic patients with SCA was somewhat unexpected given the higher prevalence of hypertension and history of stroke, particularly among women. Lower rates of screening for atrial arrhythmias among Hispanic compared with White patients may partly explain these findings.

The findings of significantly lower SCA rates among Asian compared with Hispanic and White individuals should be evaluated further. Because the Asian subgroup can be heterogeneous depending on world region of origin, these studies will need to be performed among larger numbers of patients with SCA. East Asian populations (Chinese, Filipino, Japanese, Korean, and Vietnamese) have lower overall proportional mortality rates from atherosclerotic cardiovascular disease compared with South Asian populations (Bangladesh, Bhutan, India, the Maldives, Nepal, Pakistan, and Sri Lanka).^17^ This variance has been attributed to a combination of differences in demographics, diet, risk factors, and health behaviors between these 2 large Asian subgroups.^17^ Our findings of lower SES in Hispanic compared with White individuals are consistent with published reports in the US Hispanic/Latino population, indicating socioeconomic disadvantage and lower access to preventive health care,^18^ which are additional factors that may increase the risk of SCA.^7,19^ A previous study reported a 30% to 80% higher incidence of SCA in areas of lower socioeconomic status in the Portland, Oregon, metropolitan area^6^ as well as across 7 other metropolitan areas in the US and Canada.^7^ Despite significantly lower SES among Hispanic compared with White individuals in the present study, as well as a higher cardiovascular risk factor burden, age-adjusted rates of SCA were not significantly different between the 2 ethnicities. There are several candidate factors offered in the literature that could contribute to this possibly protective effect against SCA in the Hispanic/Latino population. Rates of smoking, also associated with an increased burden of SCA,^20^ are lower in the Hispanic/Latino population.^21^ There are dietary patterns that could be unique to Hispanic/Latino individuals, including a higher intake of legumes and fruit, that contribute to reduced oxidative stress and benefits for endothelial function.^22,23^ Culturally, a higher prevalence of social and familial support,^24^ resulting in reduction of psychosocial stressors/triggers for SCA, could also contribute to protection in Hispanic/Latino individuals.^25^ These as well as other yet unidentified factors warrant further detailed investigation. Furthermore, although incidence rates were equivalent to those noted in the White population, the burden of SCA among Hispanic/Latino individuals still needs to be addressed with improved methods of prediction and prevention. The differences in SCA risk factors suggest that targeted, race/ethnicity-specific reduction of risk factors could further reduce SCA burden.

Our findings related to equivalence of SCA resuscitation outcomes between 3 racial/ethnic groups are also important from both public health and societal perspectives. Key factors that influence survival following resuscitation, such as response time, bystander CPR, and presenting rhythm during SCA, were similar across the groups, resulting in similar survival outcomes. Although these findings may not be generalizable, they provide data suggesting a positive role of community participation in survival from SCA, irrespective of ethnicity. Our results are in contrast to the sparse previously published data on SCA outcomes in Hispanic/Latino individuals. In the nationwide CARES registry (>31 000 patients with SCA, 2005-2010), Hispanic individuals with SCA were less likely to receive bystander CPR (33.7%) than White individuals (40.7%).^26^ In a statewide registry in Arizona (approximately 4800 patients, 2010-2012), SCAs in predominantly (>80%) Hispanic neighborhoods were less likely than those in predominantly (>80%) White neighborhoods to receive bystander CPR, have shockable rhythms, and survive to hospital discharge,^27^ although this study did not report individual ethnicity. More data are available for Black individuals, who are less likely to have a shockable rhythm and receive CPR, and in most reports, more likely to have lower survival rates.^28,29,30,31^

### Limitations

This study has limitations. US Census data (2018) indicate that 89% of Hispanic/Latino individuals residing in Ventura County are of Mexican or Mexican American background. Thus, it is likely that most Hispanic patients with SCA included in our study are also of Mexican background. However, Hispanic communities in the US have diverse backgrounds,^32^ with different patterns of migration and acculturation, all of which may affect health behaviors, risk, and outcomes.^33^ In addition, there is substantial genetic diversity among Hispanic groups based on region of origin, which may also be associated with risk of SCA.^34^ Therefore, our results may not be generalizable to Hispanic/Latino populations living in other regions of the US. Only 2 Hispanic individuals identified as of Black race, precluding analysis of potential differences between Black and non-Black Hispanic cohorts. The Asian population in Ventura County is relatively small and may not be representative of Asian groups in other areas of the US. In addition, the number of patients with SCA who were of Black or other race was small and precluded detailed evaluation of their clinical profile before SCA; owing to small numbers, incidence rates calculated for these groups should be interpreted with caution.

## Conclusions

In this prospective multiethnic investigation of SCA in a single large US community, we observed equivalent age-adjusted incidence rates of SCA in Hispanic, White, and Black individuals and lower rates of SCA among Asian individuals. Survival outcomes following resuscitation were equivalent in the Asian, Hispanic, and White groups, despite lower socioeconomic status in Hispanic individuals. However, the clinical risk profile of Asian and Hispanic individuals was significantly different from that of their White counterparts, and sex-specific differences were also identified. These findings highlight the need to further explore ethnicity-specific differences in SCA risk.
